# Revealing elasticity of largely deformed cells flowing along confining microchannels†

**DOI:** 10.1039/c7ra10750a

**Published:** 2018-01-03

**Authors:** Shuhuan Hu, Ran Wang, Chi Man Tsang, Sai Wah Tsao, Dong Sun, Raymond H. W. Lam

**Affiliations:** Department of Mechanical and Biomedical Engineering, City University of Hong Kong Hong Kong shuhuanhu2-c@my.cityu.edu.hk rhwlam@cityu.edu.hk +852-3442-0172 +852-3442-7174 +852-3442-8577; School of Biomedical Sciences, University of Hong Kong Hong Kong; City University of Hong Kong Shenzhen Research Institute Shenzhen China; Centre for Robotics and Automation, City University of Hong Kong Hong Kong; Centre for Biosystems, Neuroscience, and Nanotechnology, City University of Hong Kong Hong Kong

## Abstract

Deformability is a hallmark of malignant tumor cells. Characterizing cancer cell deformation can reveal how cancer cell metastasizes through tiny gaps in tissues. However, many previous reports only focus on the cancer cell behaviors under small deformation regimes, which may not be representative for the behaviors under large deformations as in the *in vivo* metastatic processes. Here, we investigate a wide range of cell elasticity using our recently developed confining microchannel arrays. We develop a relation between the elastic modulus and cell shape under different deformation levels based on a modified contact theory and the hyperelastic Tatara theory. We demonstrate good agreements between the model prediction and experimental results. Strikingly, we discover a clear ‘modulus jump’ of largely deformed cells compared to that of small deformed cells, offering further biomechanical properties of the cells. Likely, such a modulus jump can be considered as a label-free marker reflecting the elasticity of intracellular components including the nucleus during cell translocation in capillaries and tissue constrictions. In essence, we perform cell classification based on the distinct micromechanical properties of four cell lines, *i.e.* one normal cell line (MCF-10A) and three cancer cell lines (MCF-7, MDA-MB-231 and PC3) and achieved reasonable efficiencies (efficiency >65%). Finally, we study the correlation between large-deformational elasticity and translocation rates of the floating cells in the microchannels. Together, our results demonstrate the quantitative analysis of the biomechanical properties of single floating cells, which provide an additional label-free physical biomarker toward more effective cancer diagnosis.

## Abstract 2

Deformability is a hallmark of malignant tumor cells.

## Graphical abstract

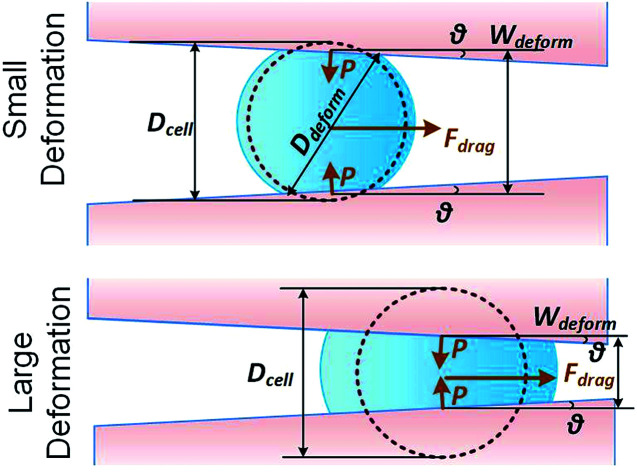

## Introduction

Cancer-related death is often caused by metastasis, in which circulating tumor cells (CTCs) disseminated in the circulation system^1^ metastasize to a second location through blood vessels (hematogenous metastasis^2^) or lymphatic vasculatures (lymphogenous metastasis^3^). These CTCs must deform and squeeze through small gaps (4–10 μm (ref. 4–6)) over vessels and tissues gaps. It has been frequently reported that metastatic cancer cells are associated with small elasticity.^7–10^ Further, such deformability is highly correlated with the invasiveness of cancer cells.^11,12^ For this reason, the quantification of cell deformability or elasticity has been suggested as a promising label-free, toxicity-free and non-destructive cell sorting and classifying method of floating cells.^10,13,14^

While novel measurement techniques of cancer cell elasticity have been frequently reported in the past two decades, most of them are based on small cell deformation and the linear elasticity assumption.^12,15–17^ In fact, the largely deformed cells should reflect more representative biomechanical properties for metastasis, as the cancer cells exhibit very large deformation during invasion and extravasation, in which both cell nucleus and cytoplasm have to deform altogether. It has been recently pointed out that nuclear deformability rather than the cytoplasmic deformability is the rate-limiting factor in the *in vivo* metastatic translocation processes.^18^ Hence, characterizing the elasticity of largely deformed cancer cells may offer a more-specific label-free marker for cancer diagnosis. To date, researchers have already provided some techniques to describe cells with large deformation. Suresh *et al.* applied the hollow shell hyperelasticity theory and a computational finite-element model to describe mechanical properties of largely deformed red blood cells during capillary vessel clogging.^19,20^ Bernick *et al.* proposed a homogenized material model to characterize the time-dependent deformation of neurons for studying traumatic brain injury (TBI) caused by large physical compressions.^21^

Various measurement techniques for biomechanical cell properties based on small deformation have been developed in recent years. However, many of these methods do not support cells with large deformation or direct deformation of the inner cell components such as the nucleus; and an analytical model is still missing for converting results from the largely deformed cells. For example, while atomic force microscopy (AFM) has been widely used for quantifying and mapping the local stiffness of adherent cells,^22^ its sharp tip with larger indentations can damage cells.^23^ Modifying AFM by replacing with a spherical tip, scanning force microscopy (SFM) circumvents the problem of cell damage.^21^ Micropipette aspiration and optical stretching are applicable for mechanical measurements of floating cells.^19,20^ These techniques can generate larger cell deformation, yet the micropipette aspiration technique measures the cortical stiffness of the cytoplasm whereas the optical trapping technique only deforms the cell membrane.^19,20,24,25^ Recently, a novel microfluidic technique utilizing a hydraulic shear force to generate hydrodynamic stretching of single floating cells has been reported of its implementation of mechanical phenotyping and deformability-based cell sorting.^26^ Though very effective and with a high throughput, the hydrodynamic stretching mainly measures the cytoplasmic elasticity. Technically, many other microfluidic methods such as the micro-pillar obstruction inside microchannels should support generating larger deformations of cells and their inner components,^8^ their applications are still limited as mechanical phenotyping as the detailed theoretical analysis and the quantification of biomechanical properties are yet unavailable.

To implement the phenotyping of large deformation of floating cells and to address the problems of experiment & theory set-up, we use our recently developed microfluidic elasticity^27^ microcytometer to quantify mechanical properties of largely deformed floating cells. Driven by the hydraulic flows, the floating cancer cells are compressed by two confining microchannel walls until the cell nuclei are also deformed. We use the analytical model extended from the Hertz–Tatara theory, *i.e.* the hyperelastic Tatara model, to analyze larger deformation of floating cells. This mechanical analysis is implemented for elucidating the utility of the large deformation properties as biomarkers for indicating the structural specialty and abnormality of the different cells types (*e.g.* normal cell line MCF-10A, cancer cell line MCF-7, MDA-MB-231 and PC3). The cell classification method based on the cell micromechanical analysis is introduced to classify the different types of cells. We further implemented a microfluidic model to predict the translocation rate of the floating cells based on the micromechanical analysis. Our micromechanical analysis of the largely deformed floating cells pointed out the possibility of utilizing these cell physical properties as biomarkers for predicting structural distortion of the CTCs, which could be useful for biopsy analysis.

## Models

**Fig. 1:**
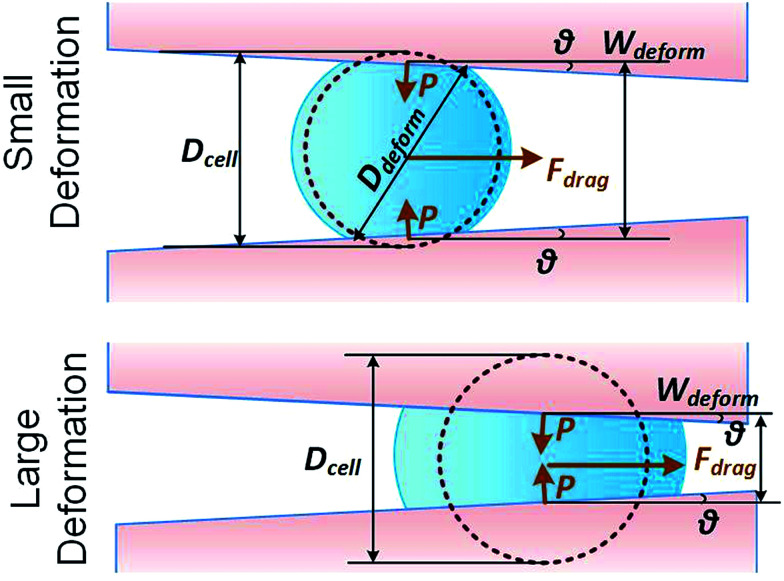
Key parameters of small and large deformation of a floating cell squeezing along a confining microchannel.

We consider a cell moving along a confining channel with inlet width *W*_in_ (=30 μm), outlet width *W*_out_ (=4 μm), channel length *L*_channel_ (=300 μm) and tapering angle *θ* (≈2.5° as tan *θ* = (*W*_in_ − *W*_out_)/*L*_channel_), as shown in Fig. 1. Cell deformation is induced by the hydraulic dragging force *F*_drag_ and the geometric confinement of the sidewalls. The sidewalls are treated with a molecular lubricant (pluoronic F127). The force balance gives *F*_compress_ = *F*_drag_/(2 sin *θ*). We have developed a hyperelastic Tatara model to describe the relation between cell stiffness and other related factors. We also estimate the cell stiffness based on the previous reported Hertz model and Tatara model for comparison as the followings.

### Hertz model

The Hertz contact model provides a general form for spherical contact under small deformation. The Young's modulus *E* is expressed as:^28^1
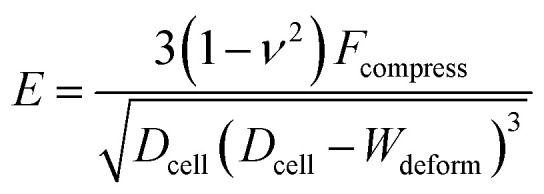
where *D*_cell_ is the cell diameter, *D*_deform_ is the cell deformed diameter, *W*_deform_ is the cell deformed width and *ν* = 0.5 is the Poisson's ratio of a cell (Fig. 1).

### Tatara model

The Tatara model extends descriptions of the Hertz model to a larger deformation regime, in which a non-spherical geometry after deformation is considered. The Young's modulus *E* obtained by the Tatara model can be expressed as:^29^2
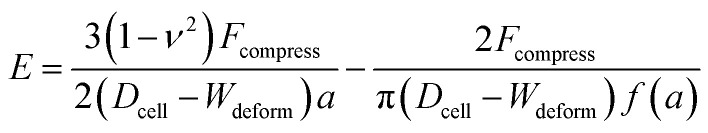
where *a* is the contact radius and *f*(*a*) is the characteristic length of the non-spherical geometry after deformation as the followings:3
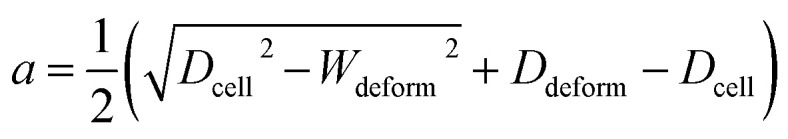
4
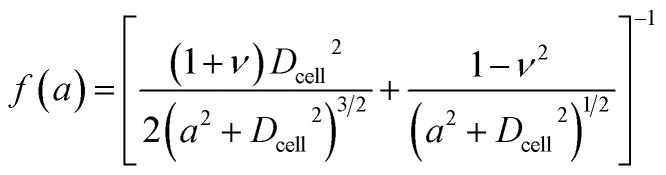


### Hyperelastic Tatara model

Hyperelasticity is well known as an intrinsic property of biologic cells; and the hyperelastic theory can describe a wide range of cell deformation.^19,20,30–32^ For a spherical hyperelastic cell, the hyperelastic Tatara's model gives an expression of the Young's modulus *E* as:^29^5
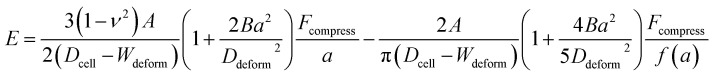
where *A* and *B* (related to hyperelastic correction) are calculated by:6
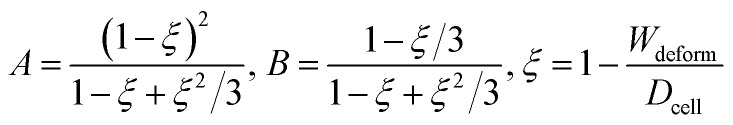
where *ξ* is the deformation of the cell.

## Materials and methods

### Fabrication

Soft lithography based on elastomeric polydimethylsiloxane (PDMS) (Sylgard-184, Dow Corning, Midland, MI) was applied to fabricate the confining microchannels.^33^ Briefly, a silicon mold master was fabricated by patterning a layer of positive photoresist (AZ5214, AZ Electronic Materials, Wiesbaden, Germany), followed by deep reactive ion etching (DRIE; STS Deep Silicon Etcher, Surface Technology Systems, Newport, UK) with a depth of 40 μm, and washing off the photoresist with acetone (Sigma-Aldrich, St. Louis, MO). The mold master was treated with vaporized (tridecafluoro-1,1,2,2-tetrahydrooctyl)-1-trichlorosilane (Sigma-Aldrich, St. Louis, MO) in a vacuum chamber to enhance the surface hydrophobicity. Afterward, the PDMS substrate with micro patterns was casted from the silicon mold. The PDMS substrate was then bonded onto a glass slide (Citoglas, Jiangsu, China) by oxygen plasma. Before the experiments, 1% (w/w) pluronic F-127 (Sigma-Aldrich, St. Louis, MO) in water was injected into the device. The device was immersed with pluronic F-127 solution for 30 min to surface treat the inner channel walls to prevent the possible cell attachments. Pluronic F-127 is a widely used biocompatible coating agent (FDA approved) to prevent cell adhesion.^34^ The pluronic F-127 treatment could reduce the friction coefficient between cell and sidewall down to 0.008.^35^

### Cell culture

Immortal human breast epithelial cells MCF-10A, malignant breast cancer cells MCF-7, invasive breast cancer cells MDA-MB-231 and prostate cancer cells PC3 were obtained from ATCC (Manassas, VA). MCF-10A cells were cultured in the Mammary Epithelial Growth Medium (MEGM; CC-3150, Lonza, New York City, NY) added with 0.4% (v/v) bovine pituitary extract (BD, Franklin Lakes, NJ), 0.1% (v/v) human epithelial growth factor (hEGF; Cell Signaling Technology, Beverly, MA), 0.1% (v/v) hydrocortisone (Sigma-Aldrich, St. Louis, MO), 0.1% (v/v) insulin (Sigma-Aldrich) and 0.1% (v/v) of a reagent mixed with 30 mg ml^−1^ gentamicin and 15 μg ml^−1^ amphotericin (GA-1000, Lonza). MCF-7 cells were cultured in a high-glucose Dulbecco's modified Eagle's medium (DMEM; Invitrogen, Carlsbad, CA) with the supplement of 10% fetal bovine serum (Atlanta Biological, Atlanta, GA), 0.5 μg ml^−1^ fungizone (Invitrogen, Carlsbad, CA), 5 μg ml^−1^ gentamicin (Invitrogen), 100 units per ml penicillin, and 100 μg ml^−1^ streptomycin. MDA-MB-231 cells were cultured in DMEM-F12 (Invitrogen) added with 10% fetal bovine serum and 100 units per ml penicillin. PC-3 cells were cultured in Roswell Park Memorial Institute media (RPMI-1640; Invitrogen) with 10% fetal bovine serum, 0.5 μg ml^−1^ fungizone, 5 μg ml^−1^ gentamicin, 100 units per ml penicillin, and 100 μg ml^−1^ streptomycin. All cells were maintained at 37 °C with 100% humidity and 5% CO_2_. All cells were cultured at 37 °C with ∼100% humidity and 5% CO_2_ in air in an incubator. 0.25% trypsin–EDTA in phosphate buffered saline (PBS; Sigma-Aldrich, St. Louis, MO) was applied to re-suspend the cells, following by centrifuge and replacement of fresh culture media. The cells were then diluted to the target cell density (∼4 × 10^4^ cells per ml) by adding additional culture media.

### Imaging and processing

A phase-contrast inverted microscope (TE300, Nikon, Tokyo, Japan) equipped with an sCMOS microscope camera (Zyla, Andor, Belfast, UK) was applied to capture high-resolution images (∼570 nm per pixel). Fluorescent images were also captured using the TE300 microscope. An open source image processing software (ImageJ; NIH, MD) and self-developed a Matlab (Mathworks, Natick, MA) script were adopted for batch processing of the microscopic images.

### Statistics

*p*-Values were calculated using the Student's *t*-test in Excel (Microsoft, Seattle, WA). Standard errors (SE) were calculated in expressing the values.

## Results

### Characterizing pressure driven cell deformation

**Fig. 2:**
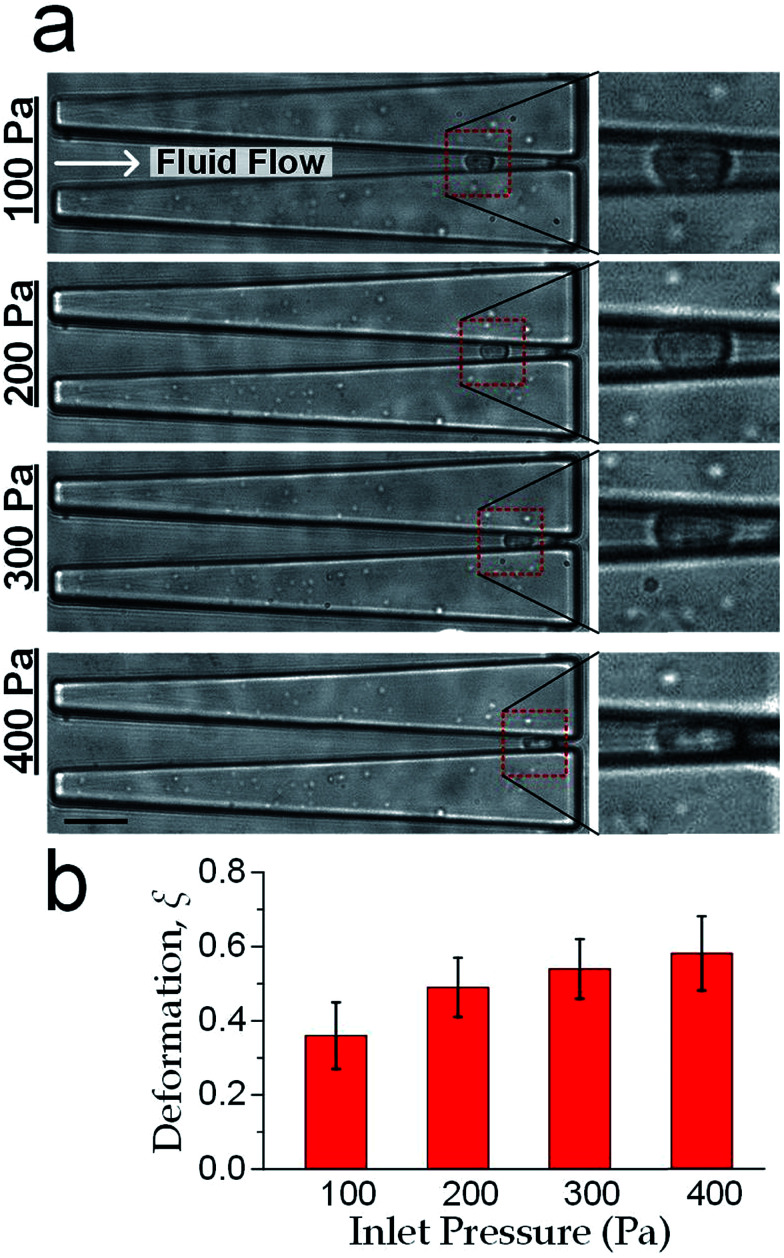
Deformation of cells (MCF-10A) as a function of inlet pressure applied at the device inlet. (a) Cell deforms in a confining channel under different inlet pressure levels (100 Pa, 200 Pa, 300 Pa and 400 Pa). Scale bar: 30 μm. (b) Statistics of the deformation level against the inlet pressure, *n* = 31. Error bars represent the standard deviation.

We adopted our previously reported elasticity microcytometer for generating different degrees of cell deformation (see ESI†).^36^ Floating cells were injected into the microfluidic device and the cells were trapped in the confining microchannels thereafter. Key parameters for the cell movement and deformation inside the microchannel are described in Fig. 1. As the hydraulic force pushed the cell forward to the narrower outlet, the cell moved along the channels and deformed until the compression forces balance the hydraulic force. Thus different compression forces *F*_compress_ acting to the cells could be regulated by different gauged inlet pressures. To estimate the *F*_compress_, the hydraulic drag force over the cell body *F*_drag_ was firstly obtained by a laminar flow simulation using COMSOL software (see ESI†), following by computing the compression force *F*_compress_ by *F*_compress_ = *F*_drag_/(2 sin *θ*). Furthermore as mentioned previously in fabrication, the microchannel walls treated with pluronic F-127 has a friction coefficient of 0.008, hence the friction force on cells was <10% of the drag force. Therefore, the cell deformation was mainly caused by *F*_compress_. In our experiments, the hydraulic dragging *F*_drag_ forces placed on the cells were 1–50 nN and the compression forces were 12–600 nN. We performed experiments with a human breast epithelial cell line (MCF-10A) to characterize cell deformation under different inlet pressures: 100 Pa, 200 Pa, 300 Pa and 400 Pa. The cell position *L* and the deformed diameter *D*_deform_ were obtained by an image analysis based on micrographs as shown Fig. 2a. The deformed width *W*_deform_ was obtained by *W*_deform_ = *W*_in_ − 2*L* tan *θ*. The cell diameter *D*_cell_ could be obtained by:7
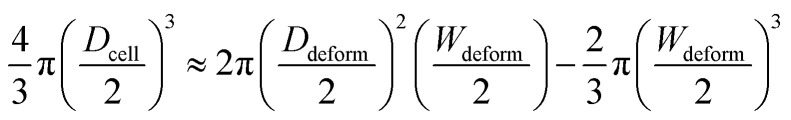


The measured cell diameter of MCF-10A is 14.83 ± SE 0.45 μm, which agrees with the reported values.^37^ The deformation *ξ* (defined as *ξ* = 1 − *W*_deform_/*D*_cell_) gradually increased with the enhancement of inlet pressure levels (Fig. 2b).

**Fig. 3:**
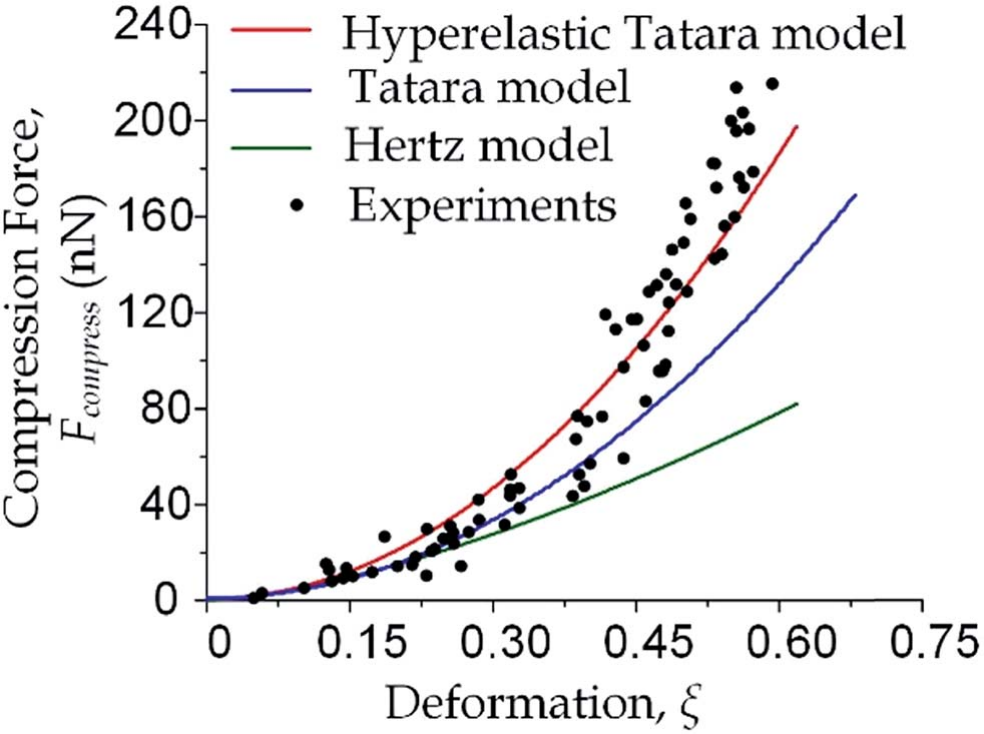
Cell deformation properties toward large deformations. The compression force *versus* cell deformation under 100 Pa and 200 Pa were plotted and compared with the predictions based on Hertz model, Tatara model and hyperelastic Tatara model respectively. The parameters (Young's modulus and cell diameter) in the predictions were obtained by the measurement results under 100 Pa.

We focused on the transition from small deformation to large deformations (*i.e.* inlet pressure from 100 Pa to 200 Pa). As shown in Fig. 3, the observations of compression force *versus* deformation was plotted (the black dots) to be compared with the predictions made by the aforementioned three models (the three lines). A deviation analysis among the three models is also available in Fig. S2.†

As the classical Hertz model is based on the assumptions of linear elasticity and small deformation,^28^ the estimated cell deformation property (the green line) deviated significantly from observations (black dots) especially when the deformation is larger than 0.3. Such apparent deviation implied that the Hertz model is not suitable for describing large cell deformations. Considering that the cell deforms into a non-spherical object, Tatara *et al.* extended the Hertz contact theory to the large deformation regime by considering a more detailed geometrical configuration as a sphere with its upper and lower sections removed as shown in Fig. 1.^29^ Though this configuration led to a closer prediction of the deformation property comparing to the Hertz model (Fig. 3, the blue line; Fig. S2a and b†), the deviations between the observations (the black dots) and the model prediction (the blue line) gradually increased with the increments of the cell deformations. Thus a more accurate model was still needed. On the other hand, our proposed hyperelastic Tatara model was obtained by further introducing the hyperelasticity modification. (Fig. S2c and d†) As biological cells are intrinsically hyperelastic,^16,17,27–29^ this model should deliver the most accurate description of the cell deformation properties comparing to the previous two models. The prediction made by the hyperelastic Tatara model (Fig. 3, the red line) matched well with the observations (the black dots) of the cell deformations, hence, the hyperelastic Tatara model gave a highly representative descriptions of cell deformations.

### Deformation-induced ‘modulus jump’

**Fig. 4:**
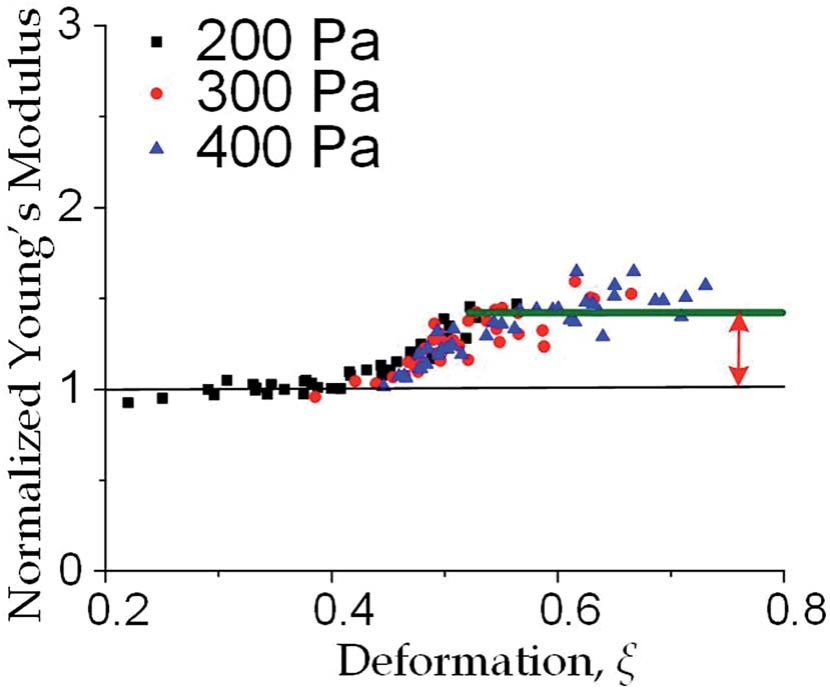
Three models for describing cell deformation of MCF-10A cells, *i.e.*, Hertz model, Tatara model and hyperelastic Tatara model. Young's modulus obtained under different deformations with the normalized modulus value scaled as unity for the level under the 100 Pa inlet pressure.

**Fig. 5:**
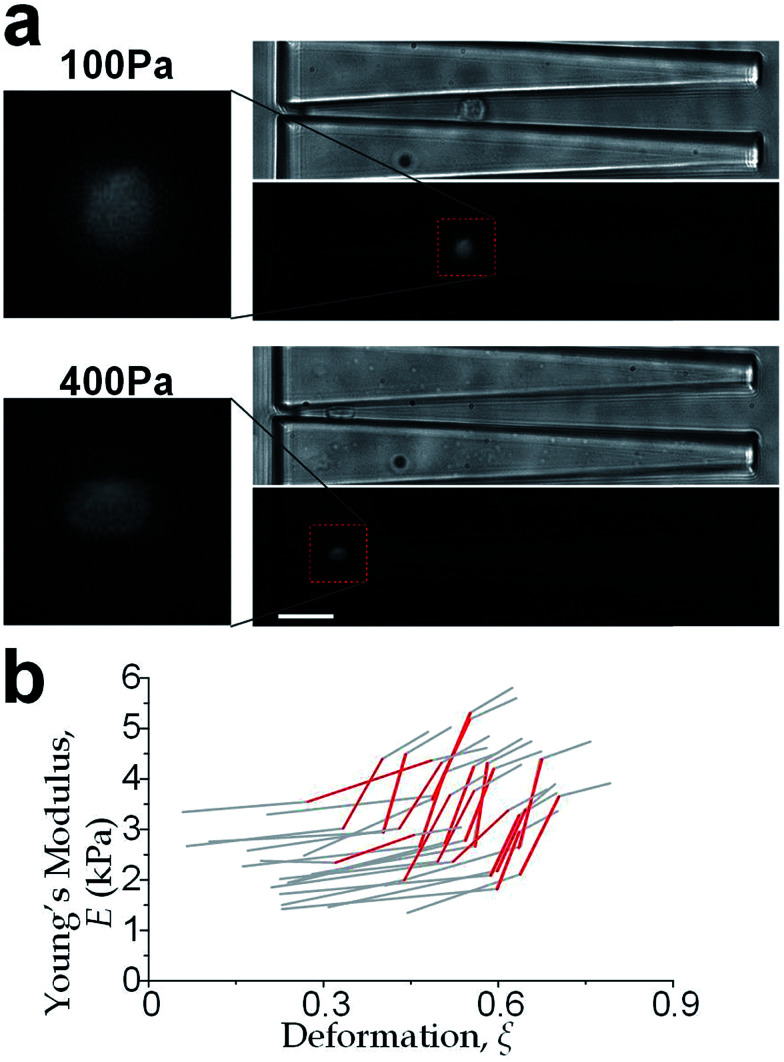
(a) Cellular and nuclear deformation of a MCF-10A cell at inlet pressure of 100 Pa (upper) and 400 Pa (lower). The cell nucleus was undeformed for the smaller deformation; whereas the nucleus deformation was created by the direct contact of the channel sidewalls when the inlet pressure is sufficient high (*e.g.* 400 Pa). Scale bar: 30 μm. (b) Deformation-induced elasticity change of an individual cell represented by each polyline. The red sections indicate the ‘modulus jump’.

We further increased the inlet pressure to 300 Pa and 400 Pa to induced nuclear deformations. When a trapped cell reached a deformation of *ξ* = 0.471 ± 0.015, we observed a ‘modulus jump’ in the Young's modulus calculated by the hyperelastic Tatara model (Fig. 4 and 5). Notably, previous research also reported such transition of the overall moduli of a core–shell polymeric composite.^38^ Likely, this change in the elasticity modulus reflects a structural heterogeneity between the shell and core parts. We examined that the cell nuclei were deformed by the direct compression from the channel sidewalls under a large enough inlet pressure (≥300 Pa) as shown in Fig. 5a. It is well known that the nuclei have been frequently shown 2–10 times stiffer than the cytoplasmic stiffness.^39–43^ It should not be a surprise to observe a ‘modulus jump’ for a largely deformed cell, as the measured Young's modulus was contributed from the nucleus stiffness rather than the cytoplasmic stiffness. This report gave two distinct empirical formulas for description the small and large deformation properties: the overall Young's moduli were given *E* = *V* × *E*_core_ + (1 − *V*) × *E*_shell_ under small deformation whereas *E* = *L* × *E*_core_ + (1 − *L*) × *E*_shell_ under large deformation, in which *V* is the core-composite volume ratio while *L* is the core-composite length ratio. Thus it is quite possible that a rigid core (the nucleus) gave a modulus jump of the floating cells and led to the transition of the Young's modulus, giving the previously frequently reported higher nuclear stiffness than cytoplasmic stiffness.^41,44–46^ We further examined that the nucleus diameter is ∼0.5 of the cell diameter (Fig. S3†); and therefore we often observed the modulus jump at a deformation ∼0.5 for most individual cells (Fig. 5b).

### Cell classification based on cell physical properties of large deformation

**Fig. 6:**
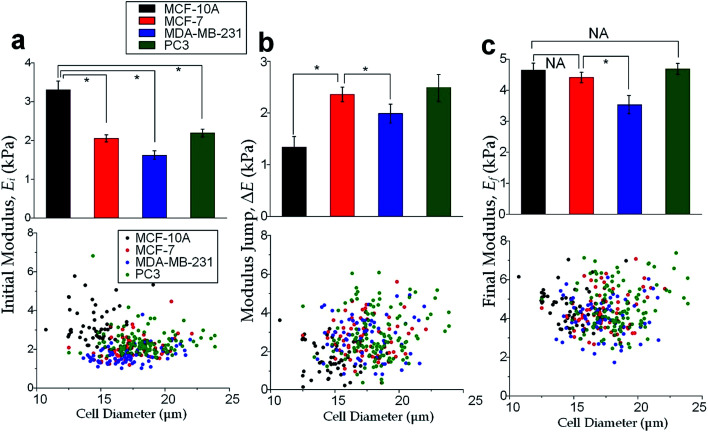
Cell mechanical properties under large deformations. Cell numbers, MCF-10A: *n* = 53, MCF-7: *n* = 73, MDA-MB-231: *n* = 67, PC3: *n* = 85. Bar charts and scattering plots of (a) the initial modulus *E*_i_ at 100 Pa, (b) the modulus jump Δ*E* between 100 Pa and 400 Pa, (c) the final modulus *E*_f_ at 400 Pa. Error bars are standard errors. * indicates *p* < 0.01.

The physical properties of large cell deformations usually imply the genetic and structural distortions.^47^ To give an example, four types of cells were used: the breast epithelial cells MCF-10A, breast cancer cells MCF-7, more invasive breast cancer cells MDA-MB-231, and prostate cancer cells PC3. A low inlet pressure (100 Pa) was applied to induce a small deformation, followed by a high inlet pressure (400 Pa) to induce a large deformation. Following the same procedures of characterizing MCF-10A cells described in the previous section, the initial moduli *E*_i_ at 100 Pa and the final moduli *E*_f_ at 400 Pa of the four types of cells were calculated by the hyperelastic Tatara model. Under small deformations, the cancerous cells (MCF-7, MDA-MB-231 and PC3) were significantly softer than the normal breast cell MCF-10A (Fig. 6a), which indicated the disruption of the cytoskeletal fibers in the cytoplasm and conformed to previous reports.^10,48^ Under large deformation, however, the MCF-7 and PC3 cells shown no significances of Young's modulus comparing to the normal cell type MCF-10A (Fig. 6c). This transition implied a stiffer or larger nuclei of MCF-7 and PC3 cells. In fact, both stiffer and larger nuclei were found in cancer cells.^49–51^ For the modulus jump (Fig. 6b), MDA-MB-231 was significantly lower than MCF-7 (or PC3), which might implicated a softer core of MDA-MB-231 than that of MCF-7 (or PC3) since the sizes of the cell body as well as the nuclei of these three types of cells were similar (see Fig. S3†). Previous research found that the more invasive cancer cells (*e.g.* MDA-MB-231) undergone epithelial–mesenchymal transition (EMT) usually contain softer nuclei.^52–54^ Hence the final modulus *E*_f_ and modulus jump Δ*E* could provide the insightful information of the physical distortion (either stiffening or enlarging) of the nuclei.

**Fig. 7:**
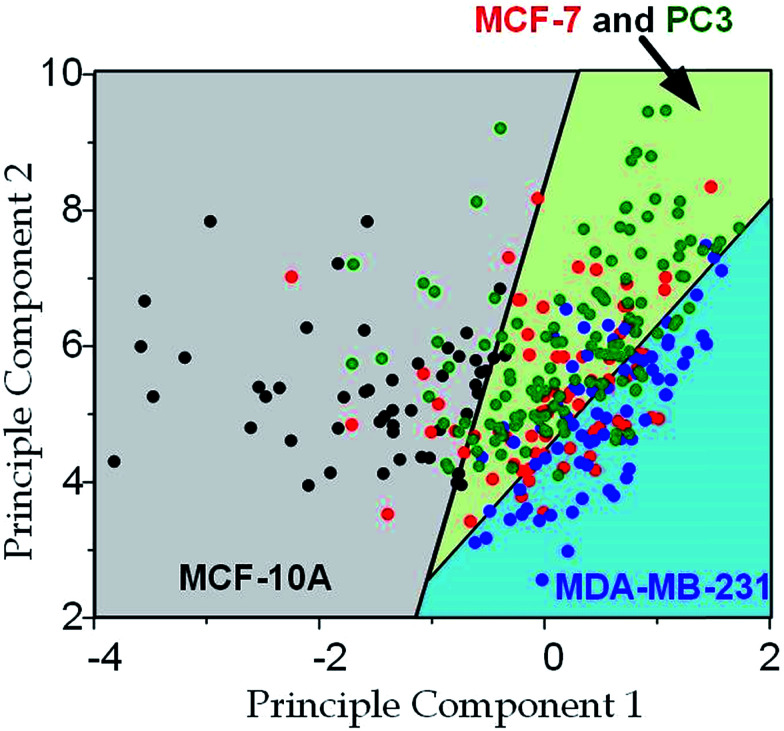
Principle component analysis and cell classification for cell classification based on cell deformation properties. Cell numbers, MCF-10A: *n* = 53, MCF-7: *n* = 73, MDA-MB-231: *n* = 67, PC3: *n* = 85; the first principle component is 0.022 × *D*_cell_ − 2.4 × *E*_i_ − 0.048 × *E*_f_ + 1.9 × Δ*E*; the second principle component is 0.046 × *D*_cell_ + 1.7 × *E*_i_ + 0.41 × *E*_f_ + 2.1 × Δ*E*.

Since the physical properties of cell deformation implied the structural abnormality of cancer cells, these physical properties could be used as biomarkers for cell classification. For demonstration, the principle component analysis (PCA)^55^ of four physical parameters (*i.e.* cell diameter *D*_cell_, initial modulus *E*_i_, modulus jump Δ*E* and final modulus *E*_f_) was applied (see Fig. 7). Next, linear discriminant analysis (LDA)^56^ was applied to classify the cells. Since MCF-7 and PC3 cells were physically undistinguishable in our experiments, the data of the two types were combined for the LDA test. These two analyses gave a relatively high efficiency for cell classification: 78.3% for MCF-10A, 65.6% for MCF-7 and PC3, 69.7% for MDA-MB-231.

### Critical pressure for a cell translocating through capillary structures

**Fig. 8:**
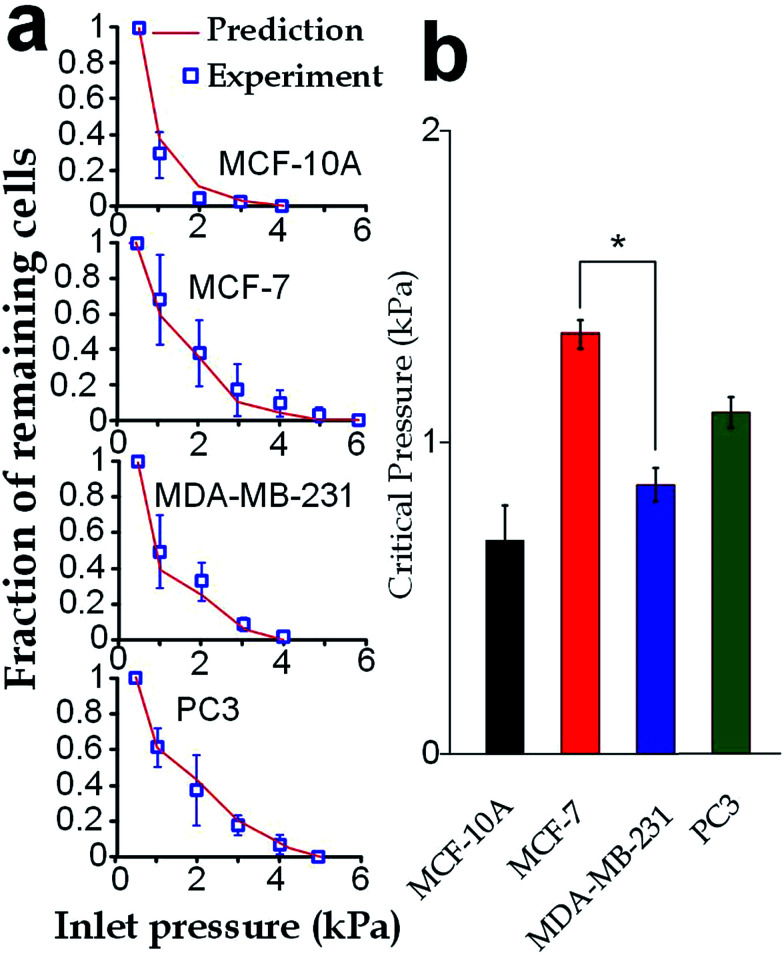
Critical pressures for flushing cells through the confining microchannels with an exit width of 4 μm. (a) Fraction of the remaining cells driven by different inlet pressures (boxes). The predicted values (lines) were computed using the hyperelastic Tatara model. (b) Critical pressures required for flushing half of the trapped cells away from the microchannels. Error bars are standard errors. Asterisk indicates *p* < 0.01.

Our elasticity microcytometer could serve as a microfluidic model for testing the capability of cancer cell translocation with nuclear deformation. For demonstration, we further increased the inlet pressures to flush away the trapped cells through the confining microchannels with an exit channel width (4 μm) significantly shorter than the nucleus diameter. After the cells got trapped in the channels, the inlet pressure was gradually increased and the number of the remaining cells was calculated, as shown in Fig. 8a. Further, predictions were made based on the deformation properties calculated by the hyperelastic Tatara model on the nucleus-deformed cells at 400 Pa. The predictions conformed very well to the experimental results, which validated the applicability of the hyperelastic Tatara model for prediction cancer cell translocating in our confining microchannel structure. Moreover, we defined and estimated the ‘critical pressure’ for half of the trapped cells passing through the microchannels. We found that, though the MDA-MB-231 cells and the MCF-7 cells had similar sizes (17.24 ± SE 0.21 μm *versus* 17.48 ± SE 0.31 μm), the critical pressure for MDA-MB-231 cells was significantly lower than the MCF-7 cells (0.91 ± SE 0.07 kPa *versus* 1.37 ± SE 0.08 kPa), which might reflect the higher capability of invasion of the MDA-MB-231 cells. Considering that the critical pressure (∼1 kPa) discovered in this work was smaller than the normal blood pressure (3.7 kPa) in capillary vessels with a typical diameter ranging 4–10 μm in human body.^4–6^ Very likely, the capillary pressure is already high enough to drive cancer cells, especially the invasive ones, through the capillaries. Therefore, the higher liquid pressure could assist cell translocation in the metastatic process.

## Conclusion

In this work, we have investigated the properties of largely deformed cancer cells using the elasticity microcytometer. We firstly extended the contact theories for small deformation and introduced the hyperelastic Tatara model for quantifying the large deformation properties of cells. Interestingly, we observed a “modulus jump” between small deformation and large deformation, which can be considered as the “rigid-core” effect as it is very likely due to the higher Young's modulus of the nucleus. Next, by applying the hyperelastic Tatara model, the distinct mechanical properties under large deformation were revealed for four different types of cells (MCF-10A, MCF-7, MDA-MB-231 and PC3). As changes in the intracellular mechanical properties can reflect the genetic and structural alterations of cancer cells, these properties can be considered as the disease-related biomarkers. Cell classification based on these biomechanical properties was also performed. Finally, we examine the relation between these properties and occurrence of the cells translocating through a confining microchannel with a narrow exit channel width (4 μm) comparable to the capillary diameter, providing some insights on the role the elasticity of largely deformed cancer cells in metastasis. This device can offer a cell characterization throughput of ∼10 cell per min, which is higher than the continuous flow optical stretcher (1 cell per min)^57^ and yet lower than the real-time deformability cytometry (6000 cells per min).^58^ In the future, we may embed microelectrode arrays along the confining microchannels for real-time cell detection and biomechanical characterization with a significant higher throughput.^59^ Importantly, the quantitative measurement technique reported in this paper can obtain elasticity and viscosity of cells in both large and small deformations; and therefore this technique would induce a more comprehensive cell characterization for more effective cancer diagnosis applications.

## Conflicts of interest

There are no conflicts of interest to declare.

## Supplementary Material

RA-008-C7RA10750A-s001
