# Supplementary material for: Revealing elasticity of largely deformed cells flowing along confining microchannels
Source: RSC Adv. 2018 Jan 3;8(2):1030–8. doi: 10.1039/c7ra10750a (PMC9076943; doi:10.1039/c7ra10750a)
Supplement: RA-008-C7RA10750A-s001 [file RA-008-C7RA10750A-s001.pdf]

Electronic Supplementary Material (ESI) for RSC Advances.
This journal is © The Royal Society of Chemistry 2017

Revealing Elasticity of Largely Deformed Cells Flowing along Confining Microchannels

Shuhuan Hu[a*], Ran Wang[a], Chi Man Tsang[b], Sai Wah Tsao[b], Dong Sun[ad] and Raymond H. W. Lam[acd*]

[a] Department of Mechanical and Biomedical Engineering, City University of Hong Kong, Hong Kong

[b] School of Biomedical Sciences, University of Hong Kong, Hong Kong

[c] Centre for Biosystems, Neuroscience, and Nanotechnology, City University of Hong Kong, Hong Kong

[d] City University of Hong Kong Shenzhen Research Institute, Shenzhen, China

* Correspondence should be addressed to S Hu (email: shuhuanhu2-c@my.cityu.edu.hk; Tel: +852-3442-7174) or RHW Lam (email: rhwlam@cityu.edu.hk; Tel: +852-3442-8577; Fax: +852-3442-0172).

Electronic Supplementary Information (ESI)

The elasticity microcytometer

The elasticity microcytometer is composed of two arrays of confining microchannels. (**Fig. S1a**) Each channel is with an inlet width W_{in} = 30 μm, outlet width W_{out} = 4 μm, channel length $L_{channel}$ = 300 μm and channel height H = 50 μm. Four bypass channels with the width of 50 μm are located at the ends of the arrays to ensure the stability of the inlet pressures of the confining channels. As shown in **Fig. S1b**, the fluidic resistance can be simplified by four resistance elements, in which $R_{in} \approx R_{out} << R_{bypass} << R_{channel}$ holds;

thus the overall fluidic resistance is always approximately the R_{bypass} whether the cells are trapped in the confining channels or not. Hence a steady inlet pressure of the microchannels is maintained in the experiments.

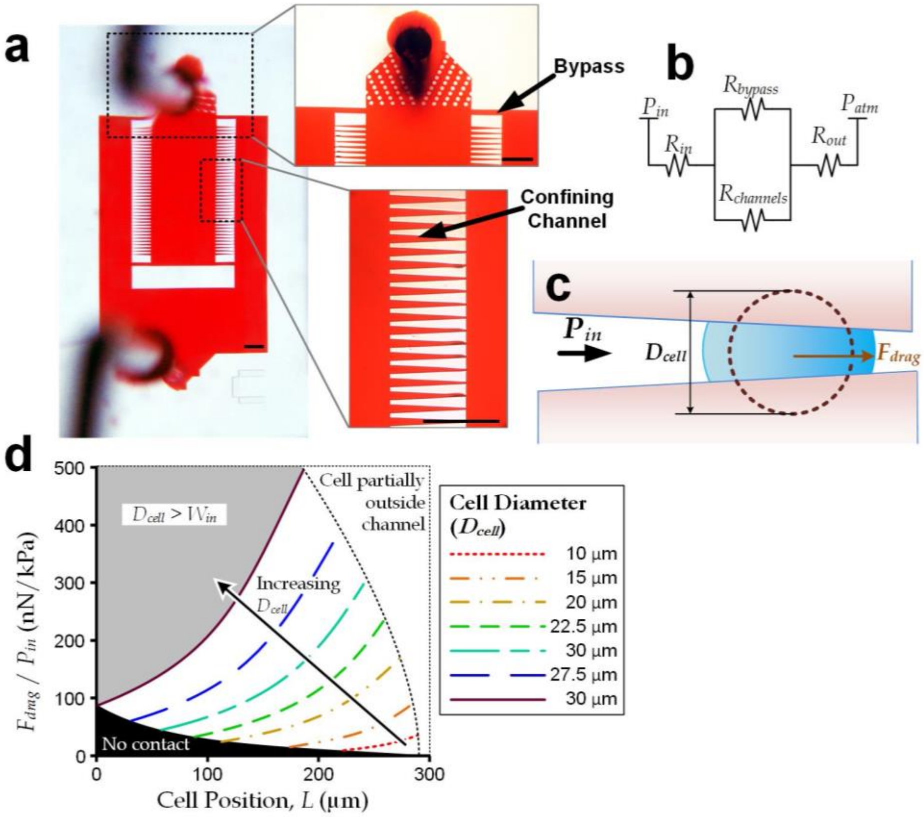

Figure S1. (**a**) Design of the elasticity microcytometer (adapted from [1]). Scale bars: 300 µm. (**b**) Diagram of the fluidic resistance of the device. (**c**) Concept figure of a cell driven by the hydraulic dragging force F_{drag}. (**d**) Comsol simulation results for calculating the hydraulic dragging force F_{drag}. The simulation results of cells with different sizes at different locations are combined (adapted from [2]).

Simulation

To obtain the hydraulic forces exerted onto the encapsulated cells, we performed finite element analysis using commercial software (COMSOL Multiphysics 4.2, Burlington, MA, USA) of multiple models of the confining microchannel structure containing an encapsulated cell with different diameters (D_{cell}) at different encapsulated position (L), in order to obtain the hydraulic pressure profile exerted onto the encapsulated cell as a function of D_{cell} and L along the channel. The position and deformed shape of a cell were

preset in the geometry of the simulation and we set the same channel dimensions (*i.e.* height $H_{channel}$, length $L_{channel}$, inlet width W_{in} and outlet width W_{out}) identical to the fabricated device in all the models, whereas in each model the deformed cell diameter (D_{deform}) was computed using **Eqn. 7**. We then obtained the resultant drag force F_{drag} by integrating the simulated pressure profile over the cell surface. By performing a series of simulations of multiple models of with different location and deformation of an encapsulated cell, we were able to obtain the relation among the drag force F_{drag}, the location L and cell diameter D_{cell}.

Deviation analysis of the three models

The whole cell strain could be calculated by $\varepsilon = \frac{1}{V}\int_V y\mathrm{d}V$, where y is the displacement of the finite elements in a deformed cell along the direction perpendicular to the confining microchannels. The whole cell strain could be expressed as:

$$\varepsilon = (1 - \frac{W_{deform}}{D_{cell}})^2 (1 + \frac{W_{deform}}{2D_{cell}}) \quad \text{(S1)}$$

The strains of MCF-10A cells were: 0.081 ± SD 0.059 at 100 Pa, 0.172 ± SD 0.073 at 200 Pa, 0.218 ± SD 0.086 at 300 Pa and 0.270 ± SD 0.109 at 400Pa.

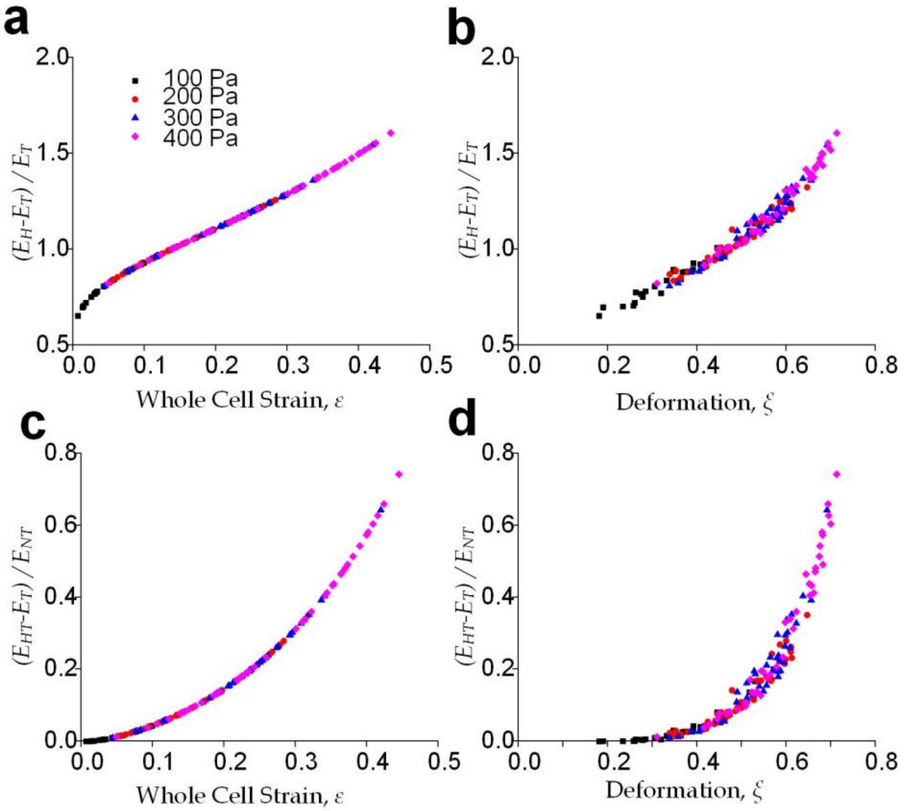

Figure S2. Deviation analysis of the three models. E_H, E_T and E_{HT} are the Young's moduli calculated by the Hertz model, the Tatara model and the hyperelastic Tatara model, respectively. (**a, b**) The deviations between and that originate from the geometric correction is almost linear; (**c, d**) The deviations between and that originate from the hyperelastic correction is strongly hyperelastic.

Statistics of the cellular and nuclear sizes

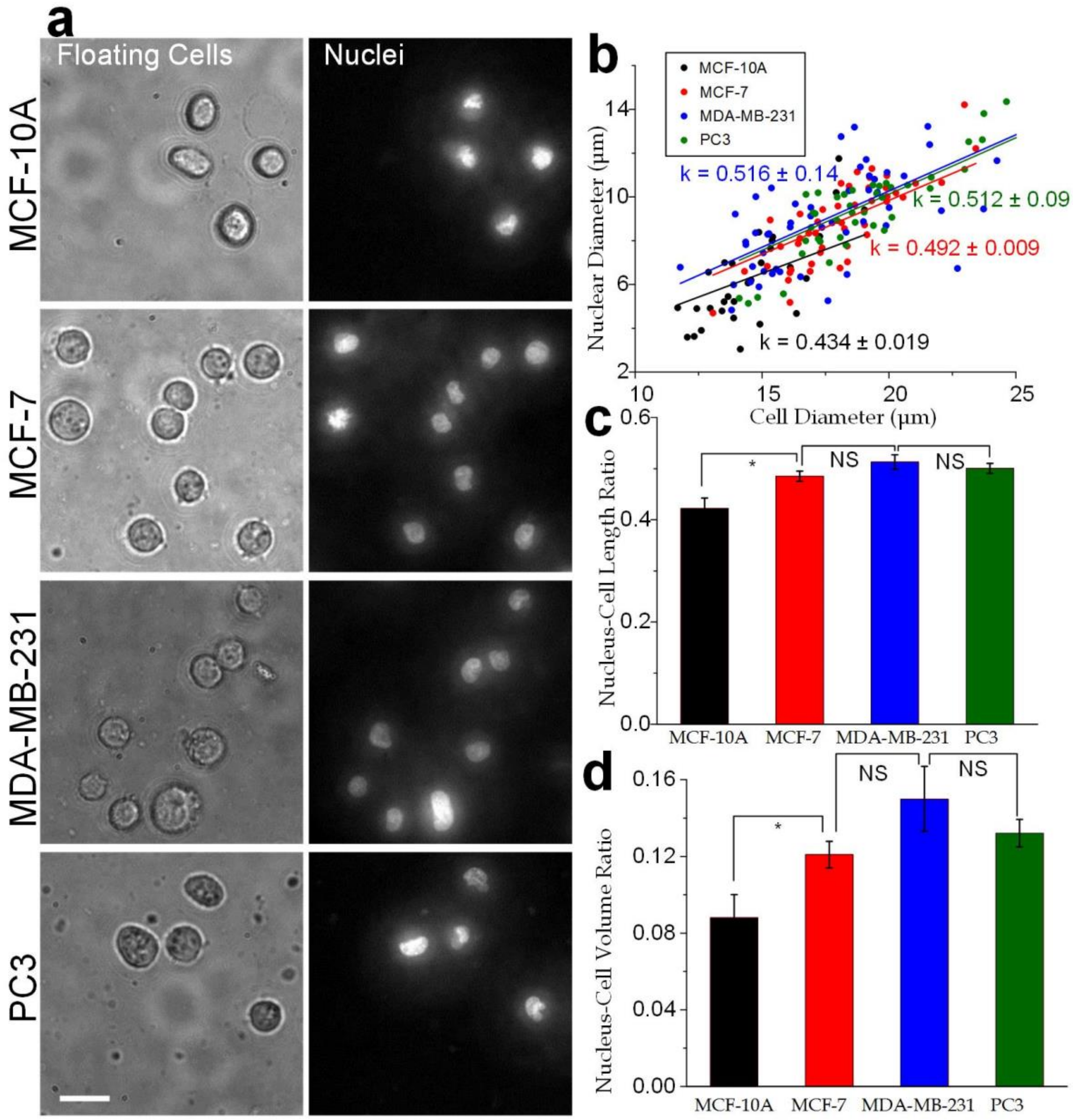

Figure S3. (**a**) The images of floating cells and the nuclei; scale bar: 20 μm. (**b**) Scattering plots of nuclear diameter versus cell diameter of MCF-10A (n = 30), MCF-7 (n = 46), MDA-MB-231 (n = 43) and PC3 cells (n = 41). The linear fitting was conducted by the least-squares method under the condition of intercept = 0. (**c**) Nucleus-cell length ratio and (**d**) nucleus-cell volume ratio of the three types of cells. Error bars: S.E.M. * indicates $p < 0.01$.

Reference

1. S. Hu, G. Liu, W. Chen, X. Li, W. Lu, R. H. Lam and J. Fu, *small*, 2016, **12**, 2300-2311.
2. S. Hu and R. H. Lam, *Microfluidics and Nanofluidics*, 2017, **21**, 68.
